# Supplementary material for: Proteomics reveals the effects of sustained weight loss on the human plasma proteome
Source: Mol Syst Biol. 2016 Dec 22;12(12):901. doi: 10.15252/msb.20167357 (PMC5199119; doi:10.15252/msb.20167357)
Supplement: Supplementary file 1 — Expanded View Figures PDF [file MSB-12-901-s001.pdf]

Expanded View Figures

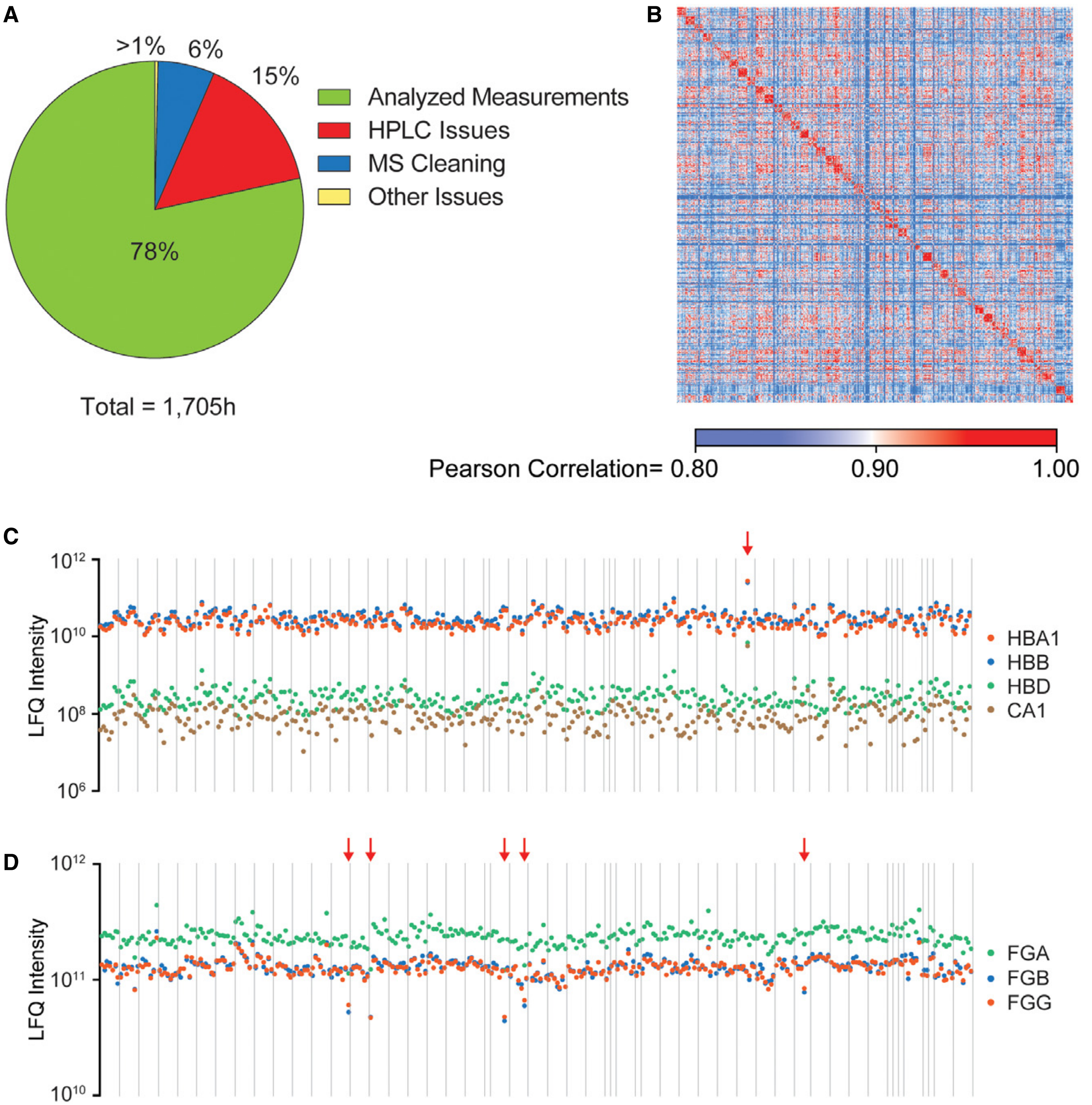

Figure EV1. MS-based measurements and sample quality.

A Total MS measuring time leading to usable raw data or lost to HPLC issues, MS cleaning, or other problems. The 6% cleaning time for the MS instrument resulted from a single cleaning instance, which, however, subsequently led to a 5-day downtime.

B Analysis of the reproducibility between samples by color-coded pairwise comparison (Pearson correlation coefficients) of 1,276 samples (without the matching library). The high reproducibility within quadruplicates and the individuals can be seen by the red diagonal, indicating high correlation.

C Levels of four highly abundant erythrocyte-specific proteins were used to assess the sample quality with regard to erythrocyte lysis. The red arrow indicates the only sample with strong erythrocyte lysis.

D Displaying the levels of fibrinogens allows the detection of (partial) blood coagulation. Five cases with decreased fibrinogen levels and therefore coagulation events are highlighted by red arrows.

A

Majority protein ID	Protein name	Gene name	-8 vs. 0	-8 vs.4	-8 vs. 13	-8 vs. 26	-8 vs. 39	-8 vs. 52
P02768	Serum albumin	ALB	+	+		+	+	+
P25311	Zinc-alpha-2-glycoprotein	AZGP1		+	+	+	+	+
P10643	Complement component C7	C7	+	+	+		+	+
D6RF35	Vitamin D-binding protein	GC	+	+		+	+	+
P07359	Platelet glycoprotein Ib alpha chain	GP1BA	+	+	+	+	+	+
O14786	Neuropilin-1	NRP1	+	+	+	+	+	+
P04278	Sex hormone-binding globulin	SHBG	+	+		+	+	+

B

Majority protein ID	Protein name	Gene name	-8 vs. 0	-8 vs.4	-8 vs. 13	-8 vs. 26	-8 vs. 39	-8 vs. 52
P02765	Alpha-2-HS-glycoprotein	AHSG		+	+	+	+	+
P02743	Serum amyloid P-component	APCS	+	+	+	+	+	+
P02652	Apolipoprotein A-II	APOA2	+	+	+	+	+	+
P04114	Apolipoprotein B-100	APOB	+	+	+	+	+	
P02655	Apolipoprotein C-I	APOC2		+	+	+	+	+
O14791	Apolipoprotein L1	APOL1		+	+	+	+	+
P01024	Complement C3	C3	+	+	+	+	+	+
P08603	Complement factor H	CFH		+	+	+	+	+
P05156	Complement factor I	CFI		+	+	+	+	+
Q92954	Proteoglycan 4	PRG4	+	+	+	+		+
P0DJI8	Serum amyloid A-1 protein	SAA1	+	+		+	+	+
P05546	Heparin cofactor 2	SERPIND1	+		+	+	+	+
P36955	Pigment epithelium-derived factor	SERPINF1	+	+	+	+	+	+

C

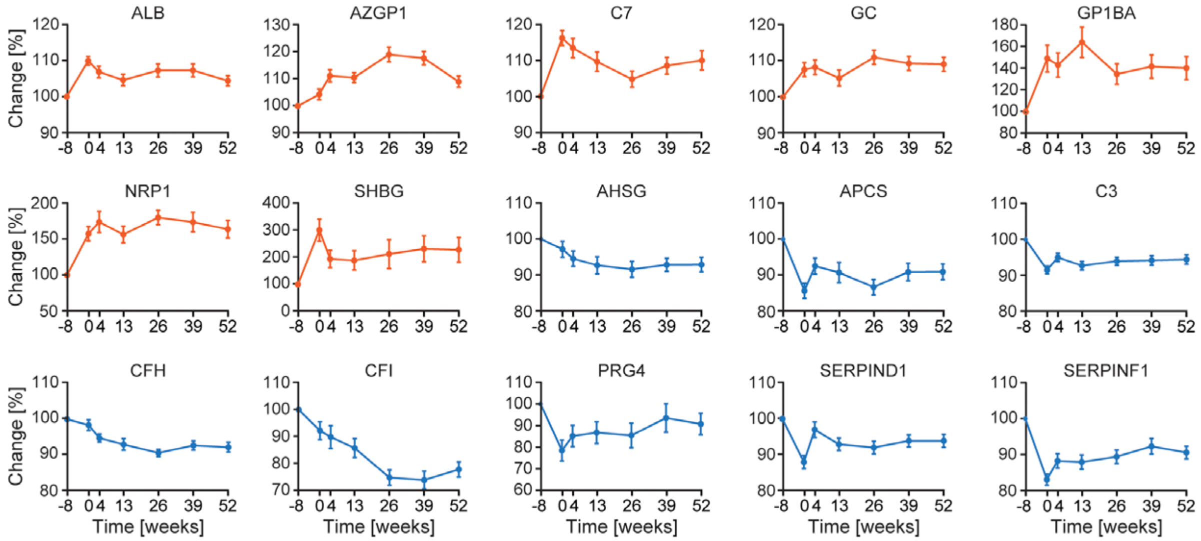

Figure EV2. Proteins with significant long-term effects due to weight loss.

A Proteins with a significant increase in at least five out of six time points, comparing before and after weight loss.

B Proteins with a significant decrease over a long time period due to weight loss.

C Long-term behavior of specific proteins. The means are plotted with SEM as error bars over time. Upregulated proteins are indicated in orange and downregulated in blue.

© 2016 The Authors

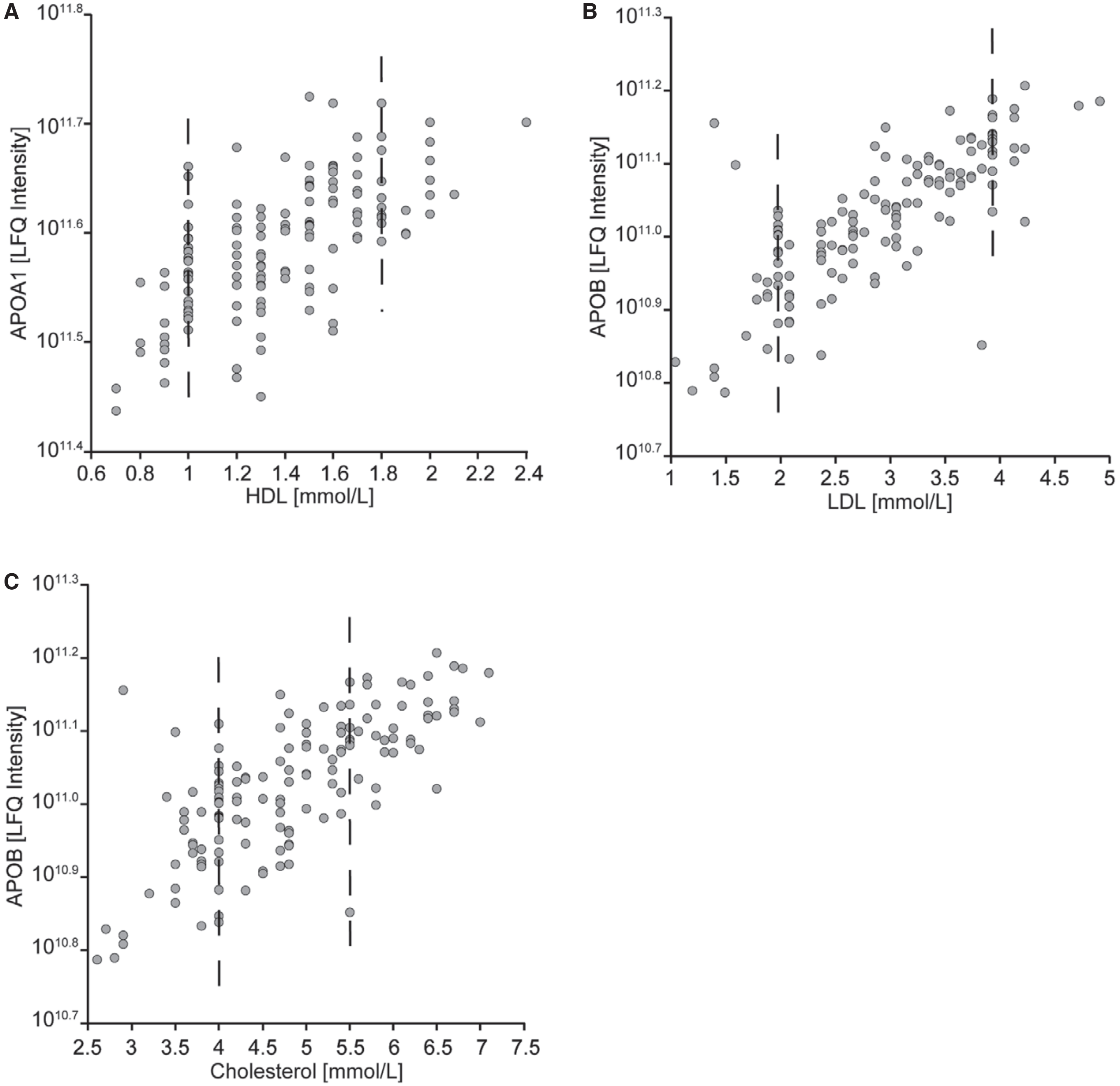

Figure EV3. MS-measured protein intensities compared to standard clinical measurements.

A Correlation of APOA1 with HDL (according to the literature each HDL particle should contain approximately one APOA1 molecule). The imperfect correlation can partially be explained by the lack of resolution of the reported HDL values, which can be seen by the non-continuous values for the individuals, resulting in vertical lines.

B MS-measured APOB intensities were correlated with LDL values (each LDL particle should contain one APOB molecule).

C Correlation of APOB intensities with cholesterol measurements.

© 2016 The Authors

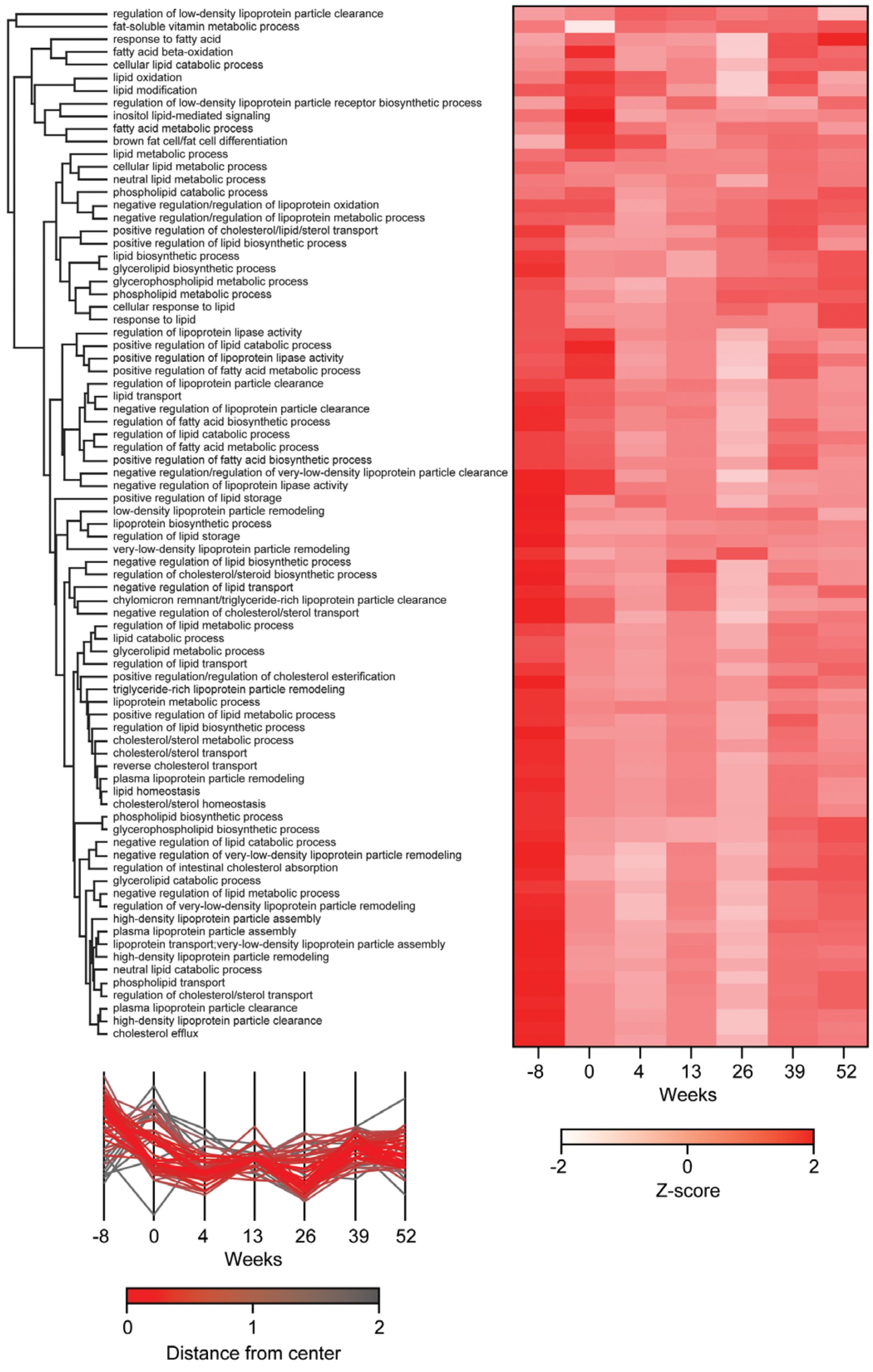

Figure EV4. Hierarchical clustering of lipid metabolism-related GOBP terms.
GOBP terms annotations were *Z*-scored over time and filtered for the keywords lipid, lipoprotein, fat, and cholesterol. Hierarchical clustering indicates lipid metabolism-related biological processes in response to weight loss and maintenance.

© 2016 The Authors
